# Factor V Leiden and MTHFR mutations as a combined risk factor for hypercoagulability in referred Patients population from Western India

**DOI:** 10.1186/1755-8166-7-S1-P28

**Published:** 2014-01-21

**Authors:** Priya Bansal

**Affiliations:** 1 Kokilaben Dhirubhai Ambani Hospital and Medical Research Institute, Four Bungalows, Andheri West, Mumbai, India

## Background

Factor V Leiden mutation is a recognized most prevalent genetic risk factor for venous thromboembolic disease. Factor V mutations, are known to potentiate the effect of MTHFR on deep vein thrombosis. The thermo labile variant of the MTHFR gene (C677T) increases the plasma homocysteine levels and hyperhomocysteneimia is a known risk factor of deep vein thrombosis. Results of studies concerning interaction of Hyperhomocysteneimia and thromobophilic risk factors like Factor V are contradictory. Some studies have shown an increased risk (10-50 times) of deep vein thrombosis because of MTHFR and FVL mutations combined, yet other studies fail to conclude similarly. We attempt to address this paradox in our study referred for DVT, Hyperhomocysteneimia and pulmonary embolism and assess the importance of the synergistic effects of FVL and MTHFR mutations.

## Material and methods

In this study we analyzed 190 (79 females and 111 males) cases referred to Kokilaben Dhirubhai Ambani Hospital and Medical Research Institute (Jan 2009 to Aug 2013) for DVT, Hyperhomocysteneimia and pulmonary embolism. MTHFR mutation was studied in 27 of the above subset. The detection of FVL mutation by PCR was studied by Restriction Fragment length polymorphism and for MTHFR (C677T & A1298C) mutations detection was done using commercially available kit.

## Results

FVL mutation was found to be present in 10% (19/190) in our study population. Of these, 18 patients were heterozygous and 1 was homozygous for this mutation. Of which 25% (19/76) patients with deep vein thrombosis were positive for variants of FVL. 74% (20/27) of the patients screened for MTHFR were found to be positive (5 for C677T, 4 were compound heterozygous & 11 for A1298C). 2 out of 4 patients who were positive for both FVL and C677T MTHFR mutations had poor prognosis and died.

## Conclusions

Our study reconfirms the Synergistic role of factor V Leiden and MTHFR (14.8%) in hypercoagulability disorders like DVT, thromboembolism and others leading to poorer prognosis.

